# Microhabitats and canopy cover moderate high summer temperatures in a fragmented Mediterranean landscape

**DOI:** 10.1371/journal.pone.0183106

**Published:** 2017-08-14

**Authors:** Gunnar Keppel, Sharolyn Anderson, Craig Williams, Sonia Kleindorfer, Christopher O’Connell

**Affiliations:** 1 School of Natural and Built Environments and Future Industries Institute, University of South Australia, Adelaide, SA, Australia; 2 School of Pharmacy and Medical Sciences, of South Australia, Adelaide, SA, Australia; 3 School of Biological Sciences, Flinders University, Adelaide, Australia; University of Oregon, UNITED STATES

## Abstract

Extreme heat events will become more frequent under anthropogenic climate change, especially in Mediterranean ecosystems. Microhabitats can considerably moderate (buffer) the effects of extreme weather events and hence facilitate the persistence of some components of the biodiversity. We investigate the microclimatic moderation provided by two important microhabitats (cavities formed by the leaves of the grass-tree *Xanthorrhoea semiplana* F.Muell., Xanthorrhoeaceae; and inside the leaf-litter) during the summer of 2015/16 on the Fleurieu Peninsula of South Australia. We placed microsensors inside and outside these microhabitats, as well as above the ground below the forest canopy. Grass-tree and leaf-litter microhabitats significantly buffered against high temperatures and low relative humidity, compared to ground-below-canopy sensors. There was no significant difference between grass-tree and leaf-litter temperatures: in both microhabitats, daily temperature variation was reduced, day temperatures were 1–5°C cooler, night temperatures were 0.5–3°C warmer, and maximum temperatures were up to 14.4°C lower, compared to ground-below-canopy sensors. Grass-tree and leaf-litter microhabitats moderated heat increase at an average rate of 0.24°C temperature per 1°C increase of ambient temperature in the ground-below-canopy microhabitat. The average daily variation in temperature was determined by the type (grass-tree and leaf-litter versus ground-below-canopy) of microhabitat (explaining 67%), the amount of canopy cover and the area of the vegetation fragment (together explaining almost 10% of the variation). Greater canopy cover increased the amount of microclimatic moderation provided, especially in the leaf-litter. Our study highlights the importance of microhabitats in moderating macroclimatic conditions. However, this moderating effect is currently not considered in species distribution modelling under anthropogenic climate change nor in the management of vegetation. This shortcoming will have to be addressed to obtain realistic forecasts of future species distributions and to achieve effective management of biodiversity.

## Introduction

Anthropogenic climate change poses a severe risk to the survival of many species [1,2]. Considerable range shifts of species [3,4] and entire ecosystems [5,6] are already being observed and it seems unlikely that many species will be able to either migrate or adapt quickly enough in response to changes in regional climate [4,5], especially those with traits conferring high sensitivity and low adaptive capacity to climate change [2]. Mediterranean landscapes are considered especially vulnerable to climate change and have been highly disturbed and fragmented by human activities [7,8,9]. However, most currently available climate data are at spatial scales too large to be relevant for organisms occupying complex habitats, resulting in much uncertainty about likely effects of climate change on the future distributions of species [10,11,12].

Macroclimatic patterns are locally modified by abiotic and biotic factors [13,14], sometimes producing microclimates buffered or decoupled from regional conditions [15,16]. Decoupling is an extreme form of buffering and involves the isolation of local environmental conditions from macroclimatic patterns [15]. For example, cold air may converge in valleys, creating cooler microclimates [15,17]. Aspect and slope influence the amount solar insolation and hence the microclimate of a location [13,18]. Furthermore, higher water-saturation in the air caused by moisture in the soil or proximity to streams and other water bodies can produce cooler microclimates during warm, and warmer microclimates during cool, periods [13,14,16]. Vegetation can reflect some solar radiation and reduce wind speeds, creating various microhabitats that have unique, often buffered, microclimates [19,20]. Similarly, some animals create burrows with unique microclimates [21,22].

The local modification of climate by abiotic and biotic factors produces a complex patchwork of microhabitats available to organisms. Species distributions are significantly affected by this fine-scale interplay of abiotic and biotic factors [16,23,24]. For example, the occurrence of smaller plants and arthropods is often dependent on shelter provided by plants [23,24]. Microclimatic conditions may also impact animal behavior as animals seek out more favorable microhabitats [25,26,27], the phenology of plants with earlier germination and senescence reported on warmer slopes in temperate climates [28,29], and the survival of both animals and plants with organisms being dependent on certain microclimatic conditions during certain stages of their life cycle [30,31]. Even anthropogenic microhabitats (such as waste items) can create microhabitats affecting the survival of insects [32]. Hence, the various microhabitats created by biotic and abiotic factors are crucial for sustaining biodiversity in most landscapes.

Because some of these microhabitats have the ability to maintain a more consistent and favorable climate compared to regional conditions, they are believed to play potentially important roles in facilitating persistence of biota under anthropogenic climate change [12,15,33] and during extreme weather events [20,34,35]. Microhabitats, such as tree hollows and leaf litter, have been shown to reduce the effects of extreme climate events and hence constitute important refuges [20,34,35]. In addition, microrefugia, habitats maintaining conditions that are more favorable for target species than regional climates over long (several generations) time periods [12,36], are increasingly considered important for *in situ* survival of species under climate change [15,16,37].

Forests and woodlands provide important microhabitats that can considerably moderate regional weather events and buffer climatic trends [12,38]. Canopy cover [14,39] and microhabitats, such as tree hollows and epiphytes [26,35], can maintain highly moderated microclimates. However, most of our climatic data are derived from meteorological stations collecting data under standard conditions (i.e., in open areas without interference by vegetation or other structures beyond a Stephenson screen), meaning that we cannot currently quantify the effects of canopy cover on microclimate [40].

In this paper we quantify the effects of canopy cover and microhabitat type on microclimate (temperature and relative humidity) in a fragmented woodland landscape in South Australia during summer. This is important because the number of days with extreme (> 35°C) heat in this state is predicted to almost triple by 2070 using regional projections under the A1F1 emission scenario, which assume extreme warming of 1.74–4.64°C globally [41,42]. Specifically, we aim to 1) document how microclimate differs in three different microhabitats; 2) identify the biotic and abiotic factors that drive the observed differences in microclimate; and 3) determine if the amount of temperature buffering afforded by the microhabitats increases with severity of heat. We predicted that the amount of microclimatic moderation would be related to topography [13,15], canopy cover [19] and the type of microhabitat [35], and that the amount of temperature buffering would increase with heat [34].

## Materials and methods

### Study site

**Fig 1:**
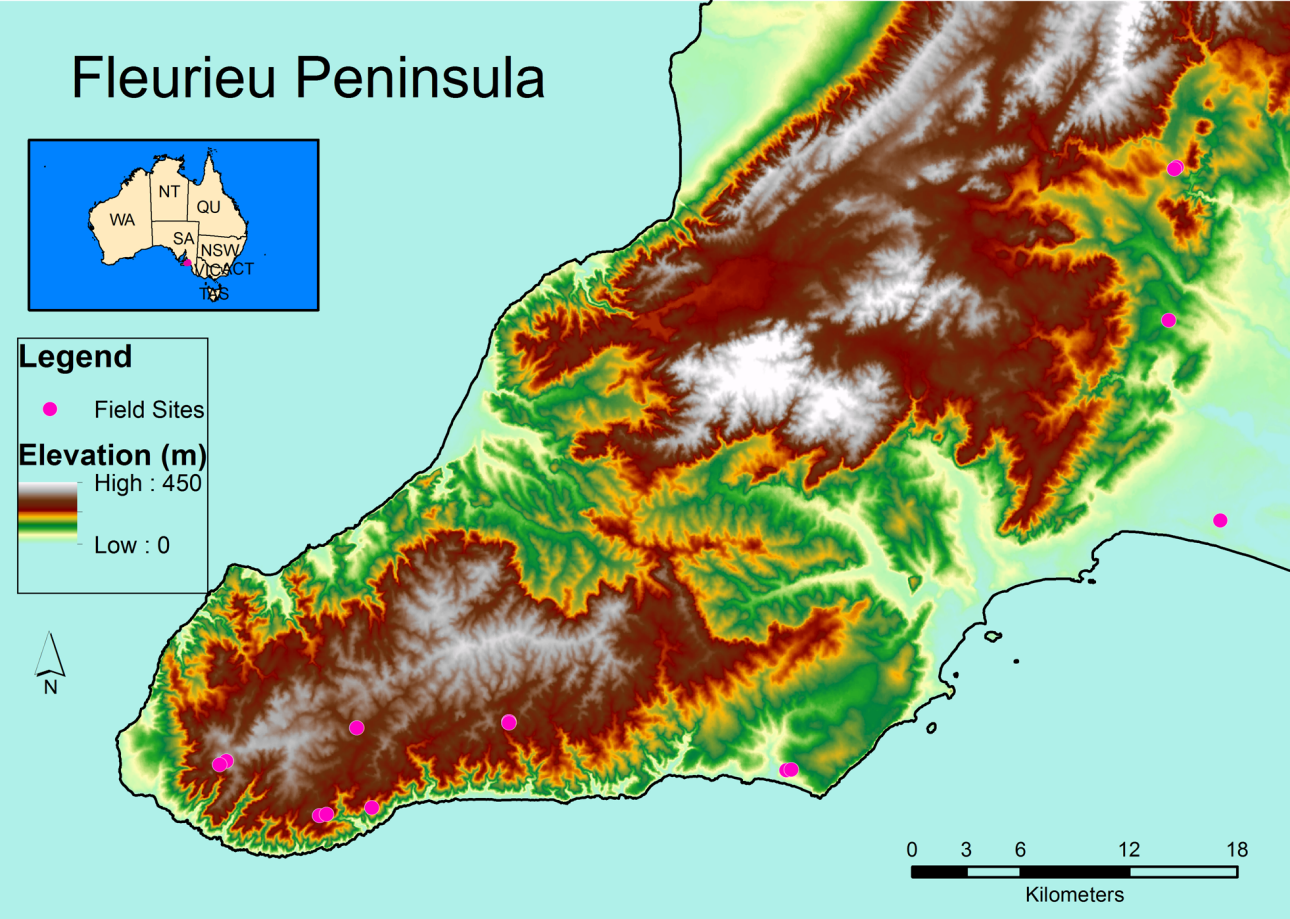
Location of the study sites on the Fleurieu Peninsula. The **s**mall inset map illustrates the location of the Fleurieu Peninsula in Australia.

The Fleurieu Peninsula is located on the south coast of Australia and has a low relief (Fig 1) with Mediterranean climate (mean annual precipitation, MAP = c. 500–1000 mm.yr^-1^; [43, 44]). It reaches to 412 m in altitude and constitutes the southernmost extension of the Mount Lofty Ranges [43,44]. The peninsula has a high concentration of species diversity, endemism and threatened species [45] and is part of the Adelaide-Kangaroo Island area, one of Australia’s centers of plant diversity and endemism [46]. Vegetation is mostly composed of eucalypt-dominated woodlands, with grassy woodlands at lower (< 200 m) and sclerophyll woodlands at higher elevations [44,47]. European settlement from 1840 had severe impacts, with near-complete removal of native vegetation, the introduction of non-native species, and an increase in erosion and fire frequency [43].

### Sampling design

**Fig 2:**
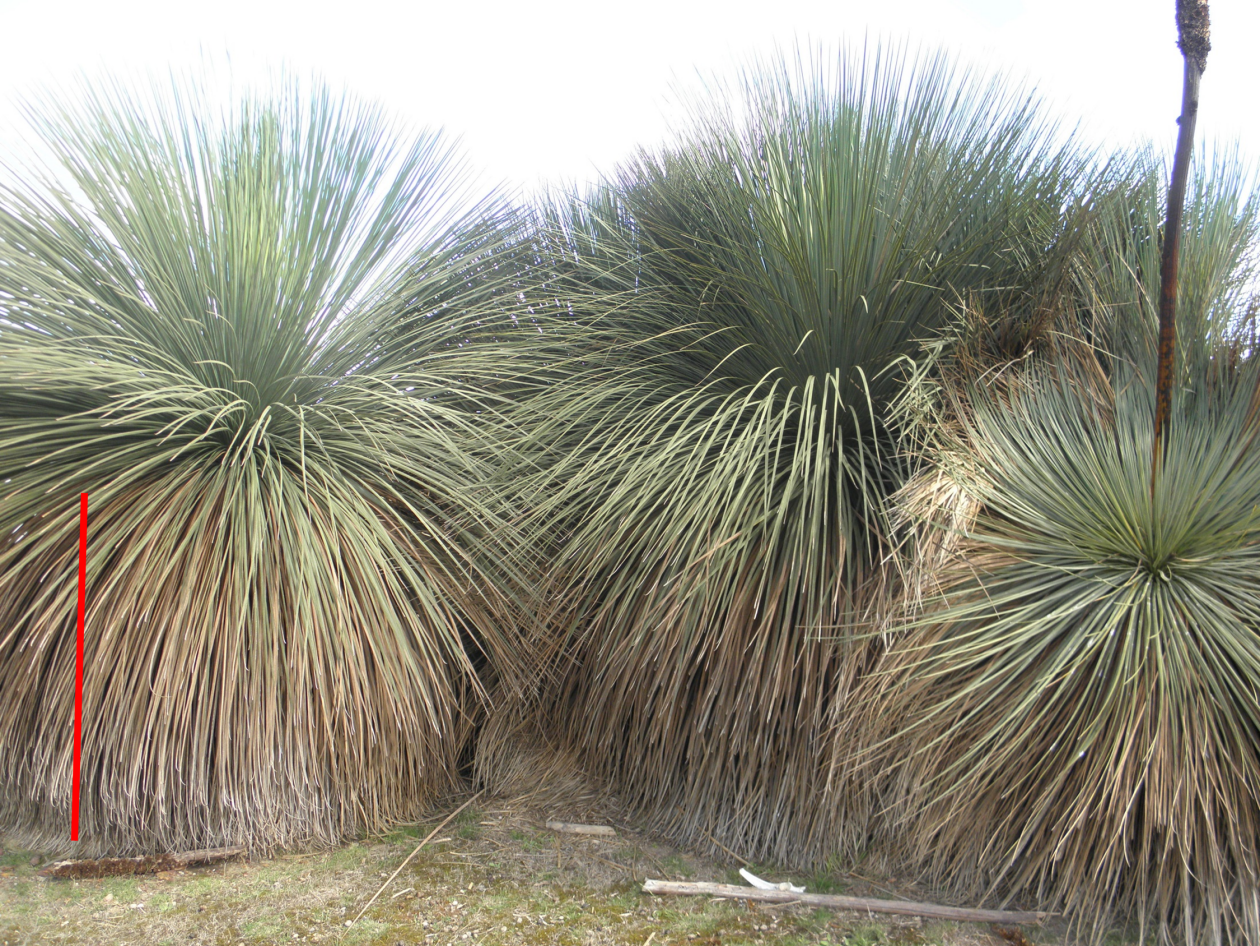
The grass tree, *Xanthorrhoea semiplana* F.Muell. (Xanthorrhoeaceae). The senescent leaves (brown colour) usually form a closed cavity, in which microsensors were placed. Red scale bar is approximately 1 m in length.

We sampled from 3 microhabitats at 14 sites across seven conservation parks (Table 1; Fig 1). Each site was sampled in two ways: (1) a circular plot with a radius of 11.28 m (area = c. 400 m^2^) was established and surveyed from 9–11 November 2015, and (2) three microhabitats (ground-below-canopy, leaf-litter, grass-tree) were selected per site and each microhabitat was equipped with up to four microsensors (*Maxim* ibuttons, DS1923) at the center of the plot to measure temperature and relative humidity (accuracy: ±0.5°C, ±5% RH), starting on 11 November 2015. Ground-below-canopy microhabitat: two sensors were placed below the canopy 50 cm above the ground on bamboo sticks on the inside of inverted, white plastic cups, which were attached with and covered with white duct tape on the upper half (S1 Fig). Leaf-litter and grass-tree microhabitat: the remaining two sensors for each site were placed inside metal tea strainers, which were also covered with white duct tape on the upper half and left uncovered in the lower half (S1 Fig). One sensors was placed in the leaf-litter (leaf-litter microhabitat) with the upper (duct tape covered) side facing upwards. When the grass tree (grass-tree microhabitat), *Xanthorrhoea semiplana* F.Muell. (Xanthorrhoeaceae), was present, another sensor was placed inside the closed cavity (see Fig 2) of this species. In addition, some microsensors were vandalised (moved out of position by humans or wildlife, recording inaccurate measurements). As a result, not every site recorded measurements for all three microhabitats (Table 1). Although we applied similar radiation shielding for all sensors and hence obtained comparable data, results would likely differ, in a consistent manner, if different radiation shielding had been used [48]. The location of each sampling site was marked directly above the ground-below-canopy sensors (plot centre) using a *Garmin GPSmap 62s*, allowing 10 minutes for equilibration of satellite readings and ensuring that a maximum accuracy of ± 3 m was reached.

**Table 1:** Locations of study sites with types of microhabitats surveyed, species richness and percentage canopy cover of fourteen 400m^2^ plots.

Park name	Site Code	Microhabitat type	Easting	Northing	Species Richness	Canopy Cover
Talisker CP	TAE	BC, LL, GT	242827	6054892	10	27
	TAI	LL, GT	242453	6054690	6	30
Deep Creek CP	DAE	BC, LL	247992	6051870	19	24
	DAI	BC, GT	248365	6051950	12	27
	DBI	BC, GT	250887	6052305	20	31
	DCI	BC, LL, GT	250047	6056733	12	30
Eric Bonython CP	EBE	BC, LL, GT	258459	6057075	12	44
	EBI	BC, LL, GT	258459	6056998	12	48
Newland Head CP	NHE	BC	273824	6054380	17	33
	NHI	BC, LL	274088	6054418	13	30
Goolwa Reserve	GRI	BC, LL	297840	6068210	14	22
Scott CP	SCE	BC, LL, GT	294982	6079284	19	16
Cox Scrub CP	CSE	BC, LL	295407	6087767	18	10
	CSI	BC, LL	295291	6087657	21	20

Easting and northing refer to UTM co-ordinates within zone 54H and were recorded in the centre of the plot. BC = ground-below-canopy microhabitat (0.5 m above the ground), GT = in the cavity formed by the senescent leaves of a grass tree, *Xanthorrhoea semiplana* F.Muell. (Xanthorrhoeaceae); LL = in the leaf litter; CP = Conservation Park.

### Vegetation

Vegetation was sampled to quantify its structural characteristics. However, we also recorded the identity and abundance of each species in each plot using a modified Braun-Blanquet scale [49] and detailed results are in S1 File. Species that could not be identified in the field were treated as morphospecies and collected for identification using literature [50,51,52] and the public reference collections of the State Herbarium of South Australia. In each plot the canopy and understorey cover of the plot were estimated visually with reference to a canopy cover chart as the average of five estimates from five locations within the plot; the center and 5 m from the center in each major direction (north, south, east and west). The maximum height of the vegetation was estimated visually with the aid of a 2 m pole in the same five locations in each plot.

### Remotely-sensed data

Remotely-sensed data was collected to derive relevant topographic and macroclimatic variables that may affect microclimate. The Geosciences Australia SRTM-derived 1-second DEM version 1.0 (http://www.ga.gov.au/elvis/) was used as the input layer for all analyses, which were implemented in ArcGIS 10.2. Solar radiation was calculated using the Solar Radiation Toolbox with the following settings; time configuration: 1 December 2015–29 February 2016, latitude: -35.5 decimal degrees, day interval: 14, hour interval: 1. This returned the potential solar radiation watt hours per square meter (WH/m^2^) for the entire study area. Slope (degrees) and aspect were derived using the ArcGIS toolbox.

The site locations were imported into *Google Earth* and used to compute the approximate shortest distances to the edge of the vegetation fragment using the ruler tool. The polygon tool was used to trace each vegetation fragment and the resulting polygon was imported into *ArcMap 10*.*1* to calculate the approximate area of each fragment. Bioclimatic variables 1 (annual mean temperature), 12 (annual mean precipitation) and 17 (precipitation of driest quarter) were obtained from relevant *WorldClim* layers (www.worldclim.org) for the 1970–2000 time period, which provide global macroclimatic data at about 1 km^2^ resolution [53]. Many of these bioclimatic variables were strongly collinear (|*r*| > 0.767; *p* < 7.4 × 10^−4^) and only mean annual precipitation (MAP) was retained for modelling of climatic parameters (see below). In addition, elevation (*r* = 0.904; *p* = 3.9 × 10^−6^; with MAP), distance to the edge (*r* = 0.770; *p* = 7.9 × 10^−4^; with solar radiation) were removed because of strong correlations.

### Microclimatic data

Data (hourly temperature and relative humidity measurements; see S2 and S3 Files for raw data) for the three summer months (1 December 2015 to 29 February 2016) was used to calculate daily minimum and maximum temperature and humidity following the protocol of the Australian Bureau of Metereology (BOM); minimum temperature being the lowest recorded temperature in the 24 hours to 9 am, and maximum temperature the highest in the 24 hours from 9 am. We calculated the average temperature/humidity as the average of the hourly temperature/humidity measurements from 12.00 am to 11.59 pm for each day. These values were averaged for the entire summer. We also calculated the variation (i.e., standard deviation) in temperature and humidity and obtained averages over the whole three months as indicators of microclimate variability and hence the microclimatic moderation provided (less variation in temperature/humidity implies more moderation). After assessing for collinearity to avoid redundant analyses, we retained average temperature, average humidity, and average variation in temperature (correlated with average minimum temperature, *r* = -0.806; *p* = 8.8 × 10^−4^; average maximum temperature, *r* = 0.830; *p* = 4.5 × 10^−4^; average temperature range, *r* = 0.923; *p* = 7.1 × 10^−6^; average variation in humidity, *r* = 0.968; *p* = 6.4 × 10^−8^).

We then determined the buffering from maximum daytime temperatures provided by the grass-tree and leaf-litter microhabitats compared to the ground-below-canopy microhabitat, which recorded the climatic conditions outside these two microhabitats. Each temperature and humidity record was assigned to either day (after sunrise and before sunset) or night based on BoM sunrise and sunset data for Victor Harbor (the largest town on the peninsula) and only daytime records retained. The hour prior to and after sunrise and sunset were removed in order to allow a settling period between the two phases. We subtracted the ground-below-canopy temperature/humidity from that recorded in the grass-tree and leaf-litter microhabitats to obtain the daytime/nighttime temperature/humidity moderation [34].

### Analyses

To identify the biotic and abiotic factors driving differences in microclimate, we used generalised linear mixed-effect models (GLMMs) with random intercepts in R.3.3.0 [54] with the *MuMIn* [55] and *lme4* [56] packages. The following variables were investigated as explanatory variables; distance to the edge of the fragment, area of the fragment, MAP (bioclimatic variable 12), solar radiation, slope, canopy cover, understorey cover and vegetation height as fixed effects and microhabitat type as a random effect. The natural logarithm of all fixed effects was used in the models to allow detection of exponential relationships with the response variables. We used a backward optimization approach, removing all non-significant (*p* > 0.05) variables from an initial model that included all non-correlated variables. Significance was tested using ANOVAs between the model including and excluding the variable. We used a pseudo-*r*^2^-value [57] to estimate the explanatory power of the model (conditional *r*^2^) and the fixed (marginal *r*^2^) and random effects (difference between conditional and marginal *r*^2^).

To determine, if the buffering of daytime temperature increased with the severity of heat, we used linear models with daytime temperature buffering (average for the three months per site) as the response variable. The average maximum temperature was used as an explanatory variable in addition to the fixed effects used in the GLMM above. Separate models were constructed for grass-tree (n = 8) and leaf-litter (n = 11) microhabitats. In addition, we regressed the amount of average daytime buffering against the maximum temperature for each day in each of the grass-tree and leaf-litter microhabitats. Linear regression lines were fitted using the method of least squares. The slopes of these lines were taken as the average increase of daytime buffering with increasing heat stress.

## Results

### Variation among microhabitats

**Fig 3:**
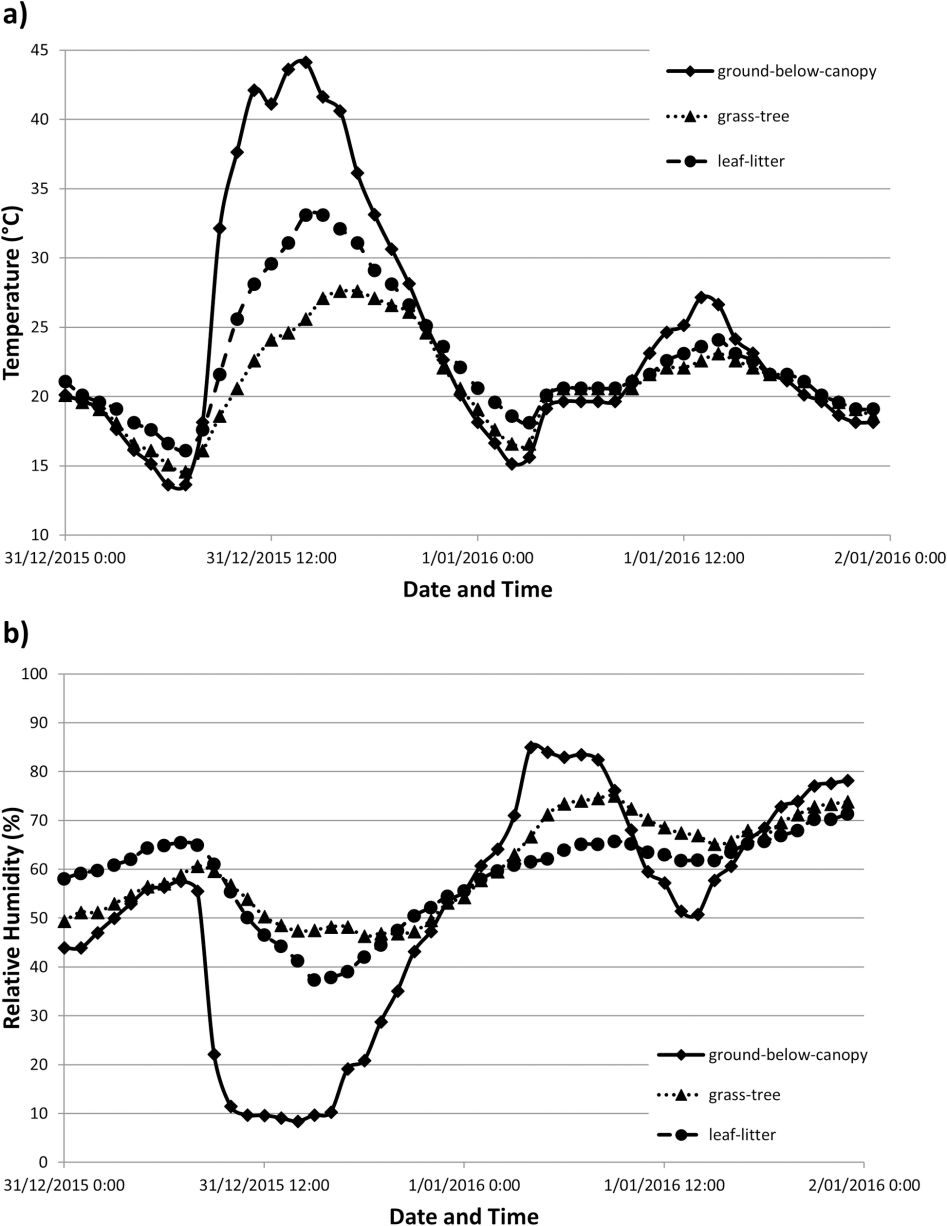
Hourly temperature and relative humidity data for a 48 hour period (from 12am on 31 December 2015 to 12 am on 2 January 2016) for microsensors in the three microhabitats at Scott Conservation Park, site SCE.

**Fig 4:**
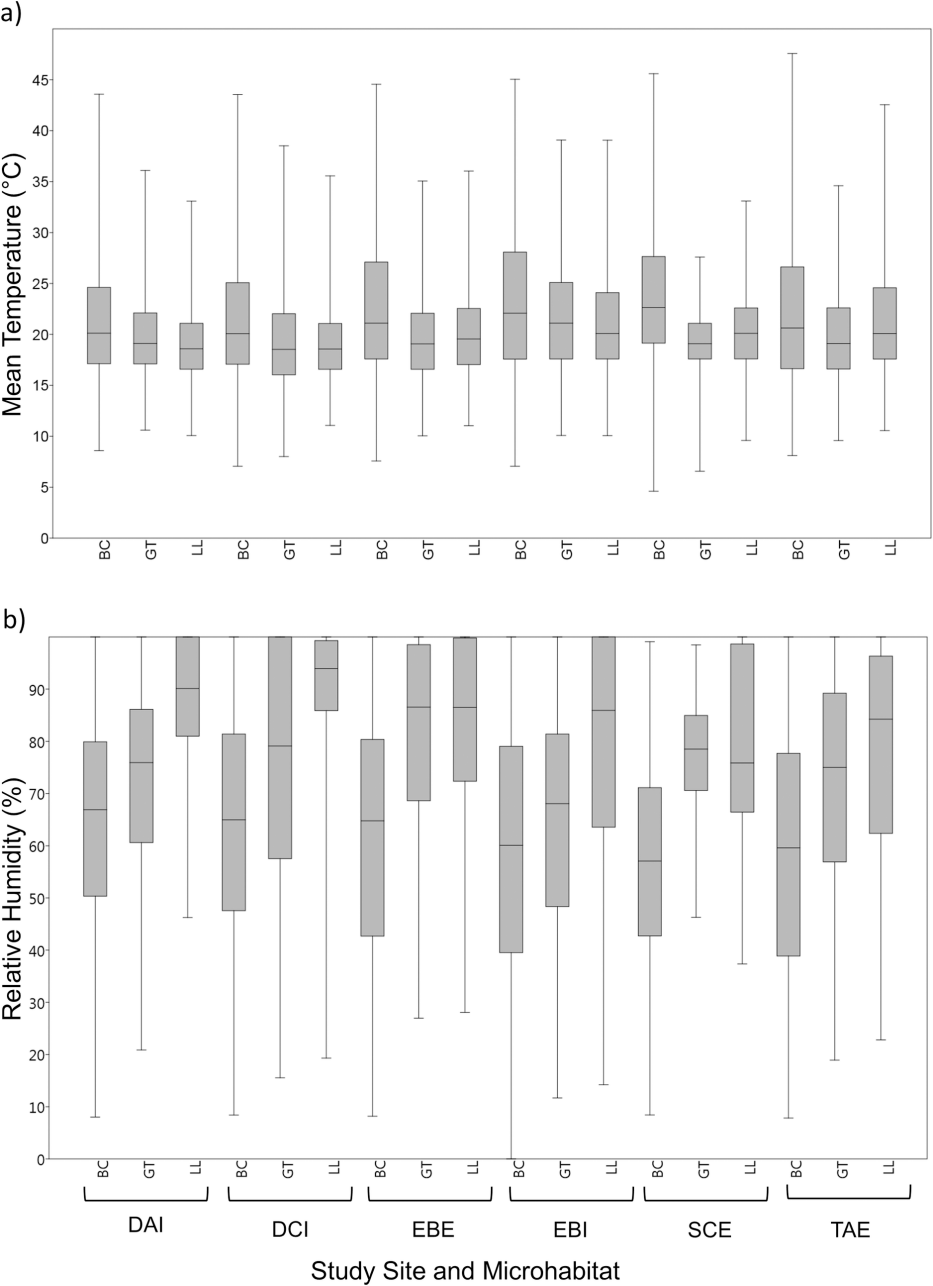
Box plots showing the median (centre lines of boxes), first and third quartiles (lower and upper box boundaries, respectively) and highest and lowest values of daytime a) temperature and b) relative humidity for the three microhabitats in six sites over three summer months from 1 December 2015 to 29 February 2016. Micohabitat codes: BC = ground-below-canopy microhabitat (0.5 m above the ground), GT = in the cavity formed by the senescent leaves of a grass tree, Xanthorrhoea semiplana F.Muell. (Xanthorrhoeaceae); LL = in the leaf litter. See Table 1 for site codes.

Temperature was higher, and relative humidity lower, during the day than at night (Fig 3). Temperature and humidity were less variable in the grass-tree and leaf-litter microhabitats compared to ground-below-canopy microhabitat, which manifested in lower temperature/ humidity ranges, lower maximum temperatures (usually 5–10°C lower; up to 14.4°C)/ higher minimum humidities, less temperature variability and slightly lower mean temperatures (Tables 2 and 3, Figs 3 and 4). Temperatures in leaf-litter and grass-tree microhabitats were strongly moderated, especially during the day (Fig 4), Average temperatures were mostly being 1–5°C cooler during the day and 0.5–3°C warmer at night than in the ground-below-canopy microhabitat (Table 2). The average humidity was lowest (mostly 0–70%) and most variable in the ground-below-canopy microhabitat, and highest (mostly 72–90%) and least variable in the leaf-litter microhabitat, with grass-tree microhabitats displaying intermediate values (Table 3, Fig 4). On average, relative humidity in the leaf-litter habitat was about 8–25% higher during the day (7–20% in the grass-tree microhabitat) than ground-below-canopy microhabitat (Table 3).

**Table 2:** Average, mean daily variation and mean daytime and nighttime moderation (*italics*: *day; night*) of temperature (°C, with standard deviation) for three microhabitats (ground-below-canopy, under grass-tree, in leaf-litter) of 14 study sites on the Fleurieu Peninsula, South Australia.

Site	Ground-below-canopy (mean, variation, range)	Grass-tree (mean, variation, range)	Leaf-litter (mean, variation, range)
CSE	20.3 (±4.2), 7.0 (±2.5), 0.5–51.0		19.5 (±3.1), 4.1 (±1.7), 6.7–36.6 *(-4*.*8±2*.*5; 3*.*6±1*.*6)*
CSI	20.4 (±4.2), 6.1 (±2.3), 3.5–48.0		
DAE	19.9 (±4.2), 4.7 (±1.9), 6.6–45.6		19.6 (±4.1), 3.7 (±1.9), 7.6–46.1 *(-1*.*1±1*.*2; 0*.*4±0*.*9)*
DAI	19.1 (±4.0), 4.0 (±2.4), 7.1–43.6	18.5 (±3.0), 2.4 (±1.2), 9.6–36.1 *(-2*.*0±2*.*0; 0*.*9±1*.*3)*	18.0 (±2.6), 2.1 (±1.1), 9.6–33.1 *(-2*.*7±2*.*3; 0*.*5±1*.*5)*
DBI	20.6 (±3.7), 4.6 (±1.6), 8.6–44.6	20.7 (±2.6), 3.5 (±1.1), 12.6–38.1 *(-1*.*8±1*.*9; 2*.*2±1*.*5)*	
DCI	19.0 (±3.9), 4.7 (±2.0), 6.6–43.6	18.0 (±3.5), 3.1 (±1.7), 8.1–38.5 *(-2*.*7±1*.*9; 0*.*6±0*.*8)*	18.1 (±2.5), 2.2 (±1.3), 11.1–35.6 *(-3*.*4±2*.*2; 3*.*0±1*.*6)*
GRI	21.3 (±3.1), 6.2 (±1.9), 6.6–50.1		21.1 (±3.9), 2.7 (±1.0), 13.1–36.1 *(-3*.*2±2*.*0; 1*.*0±1*.*3)*
EBE	19.6 (±4.1), 5.0 (±2.0), 7.8–44.6	18.4 (±3.1), 2.5 (±1.3), 10.0–35.1 *(-3*.*2±2*.*0; 1*.*0±1*.*3)*	18.8 (±2.8), 2.7 (±1.2), 10.5–36.0 *(-2*.*8±2*.*1; 1*.*3±1*.*3)*
EBI	20.0 (±4.0), 5.4 (±2.0), 7.1–45.1	19.8 (±3.5), 3.7 (±1.6), 9.6–39.1 *(-1*.*6±1*.*5; 1*.*4±0*.*8)*	19.3 (±2.8), 3.5 (±1.4), 9.6–39.1 *(-2*.*5±2*.*1; 1*.*1±1*.*4)*
NHE	20.5 (±3.3), 4.5 (±2.1), 8.1–45.6		
NHI	21.4 (±3.5), 5.3 (±1.9), 9.6–45.1		21.1 (±2.7), 3.3 (±1.5), 13.1–40.1 *(-2*.*1±1*.*7; 1*.*7±1*.*5)*
SCE	20.3 (±3.9), 5.6 (±2.2), 4.6–45.6	17.9 (±2.5), 2.5 (±1.0), 6.6–27.6 *(-5*.*2±3*.*2; 0*.*7±0*.*9)*	19.1 (±2.6), 2.6 (±1.2), 9.6–33.1 *(-3*.*8±2*.*1; 1*.*7±1*.*5)*
TAE	19.5 (±4.7), 4.7 (±1.8), 7.1–47.6	18.5 (±3.5), 2.7 (±1.1), 9.1–34.6 *(-2*.*5±1*.*8; 0*.*6±1*.*2)*	19.4 (±2.9), 3.8 (±1.4), 9.6–42.6 *(-1*.*0±2*.*4; 0*.*7±1*.*9)*
TAI		19.3 (±4.6), 3.3 (±1.7), 8.6–40.1 *(-1*.*2±1*.*2; 0*.*8±0*.*8)*	20.5 (±3.8), 4.1 (±1.9), 9.6–46.1 (0.5*±2*.*3; 1*.*6±1*.*9)*

See Table 1 for study site codes. Blank cells imply that no data is available.

**Table 3:** Average, mean variation and mean daytime and nighttime moderation (*italics*: *day; night*) of percentage relative humidity (with standard deviation) for three microhabitats (ground-below-canopy, under grass-tree, in leaf-litter) of 14 study sites on the Fleurieu Peninsula, South Australia

Site	Ground-below-canopy (mean, variation, range)	Grass-tree (mean, variation, range)	Leaf-litter (mean, variation, range)
CSE	66.3 (±12.2), 24.0 (±6.5), 7.2–100.0		82.2 (±13.7), 6.6 (±4.5), 31.7–100.0 *(28*.*0±9*.*6; -0*.*6±9*.*7)*
CSI	66.1 (±13.6), 19.0 (±5.8), 7.9–99.9		
DAE	68.1 (±17.1), 15.3 (±6.3), 6.2–100.0		72.2 (±20.4), 9.4 (±6.8), 10.2–100.0 *(8*.*1±10*.*8; -1*.*18±5*.*5)*
DAI	70.9 (±16.3), 13.2 (±6.3), 8.0–100.0	75.3 (±14.6), 6.9 (±3.9), 20.9–100.0 *(9*.*0±6*.*8; -2*.*1±8*.*7)*	89.9 (±10.4), 2.4 (±2.9), 46.2–100.0 *(25*.*0±11*.*0; 10*.*7±12*.*2)*
DBI	66.9 (±15.1), 15.2 (±5.4), 9.9–100.0	68.9 (±16.1), 7.1 (±3.3), 15.4–100.0 *(7*.*2±9*.*0; -5*.*0±10*.*2)*	
DCI	71.8 (±15.5), 15.7 (±6.5), 8.4–100.0	78.0 (±20.4), 6.9 (±6.3), 15.5–100.0 *(12*.*9±11*.*7; -2*.*8±10*.*4)*	90.5 (±12.1), 4.4 (±4.7), 19.3–100.0 *(25*.*1±15*.*0; 10*.*0±12*.*0)*
GRI	65.2 (±11.1), 18.3 (±5.2), 5.9–99.9		81.3 (±14.9), 3.5 (±2.7), 36.9–100.0 *(25*.*7±11*.*1; 2*.*8±12*.*4)*
EBE	69.7 (±16.6), 15.4 (±6.4), 8.2–100.0	88.3 (±16.2), 4.9 (±4.7), 27.0–100.0 *(20*.*3±9*.*3; 4*.*3±9*.*5)*	85.3 (±14.7), 4.4 (±4.2), 28.1–100.0 *(22*.*2±9*.*0; 6*.*3±9*.*7)*
EBI	68.5 (±16.8), 16.9 (±6.7), 0.0–100.0	69.8 (±17.0), 11.0 (±5.4), 11.7–100.0 *(5*.*9±5*.*2; -5*.*0±5*.*3)*	82.6 (±17.6), 6.8 (±5.9), 14.2–100.0 *(20*.*8±10*.*5; 4*.*9±9*.*8)*
NHE	67.8 (±12.4), 15.0 (±6.0), 7.8–100.0		
NHI	69.5 (±16.5), 14.7 (±7.0), 6.1–100.0		72.9 (±16.4), 6.6 (±3.5), 21.6–100.0 *(9*.*7±7*.*2; -5*.*6±10*.*6)*
SCE	65.8 (±13.2), 16.1 (±5.3), 8.4–99.1	77.5 (±8.7), 4.0 (±2.2), 46.3–99.4 *(20*.*7±8*.*6; -0*.*9±5*.*2)*	81.9 (±15.2), 3.8 (±2.8), 37.4–100.0 *(23*.*5±10*.*3; 5*.*9±9*.*7)*
TAE	66.2 (±19.6), 15.4 (±6.6), 7.8–100.0	75.5 (±18.3), 7.9 (±5.4), 22.8–100.0 *(13*.*7±9*.*1; 3*.*4±9*.*5)*	81.6 (±14.8), 8.1 (±5.7), 22.8–100.0 *(20*.*3±11*.*7; 8*.*8±14*.*8)*
TAI		68.8 (±20.4), 9.6 (±5.9), 11.4–100.0 *(6*.*0±6*.*0; -1*.*9±6*.*1)*	82.1 (±15.9), 6.5 (±5.6), 24.5–100.0 (21.4*±12*.*6; 8*.*5±13*.*2)*

See Table 1 for study site codes. Blank cells imply that no data is available.

### Drivers of microclimatic variation

Mean annual precipitation (MAP) had a strongly significant (*p* = 6.9 × 10^−4^) negative relationship with average temperature and together with vegetation height explained about 36% of the variation (Table 4). Microhabitat type explained an additional 14%, meaning that the complete model explained about half of the variation in average temperature. None of the fixed effects strongly predicted average humidity. Although vegetation height was a significant (*p* = 0.01) predictor, it only explained < 6% of the total variation. However, the type of microhabitat type explained about 70% of the variation in average humidity and the average variation in temperature (Table 4). In addition, canopy cover (*p* = 1.2 × 10^−3^) and the area of a vegetation fragment (*p* = 0.02) had significant negative relationships with the average variation in temperature, together explaining almost 10% of the total variation (Table 4).

**Table 4:** Best generalised linear mixed-effect models (GLMMs) explaining average temperature, average humidity, and average variation in temperature with microhabitat type as the only random effect, showing only significant fixed effects.

	Av. Temperature (°C)			Av. Humidity (%)			Av. Variation in Temperature (°C)		
	Slope	Chi	*p*-value	Slope	Chi	*p*-value	Slope	Chi	*p*-value
log(MAP)	-2.85	7.96	4.8 × 10^−3^						
log(Height)	-0.68	5.38	0.02	6.56	4.13	0.01			
log(Canopy)							-0.13	10.57	1.5 × 10^−3^
log(Area)							-1.17	5.42	0.02
Marginal *r*^2^	0.359			0.056			0.095		
Conditional *r*^2^	0.497			0.762			0.794		

MAP = mean annual precipitation (mm), Height = vegetation height (mm), Canopy = canopy cover (%), Area = fragment area (m^2^). Significance of variables was tested at the 5% level using ANOVA of the model including and not including the respective term. Conditional and marginal *r*^2^ are pseudo-*r*^2^-values [57], with the conditional *r*^2^ estimating the explanatory power of the entire model and the marginal *r*^2^ that of the fixed effects.

### Temperature buffering with increasing heat

**Fig 5:**
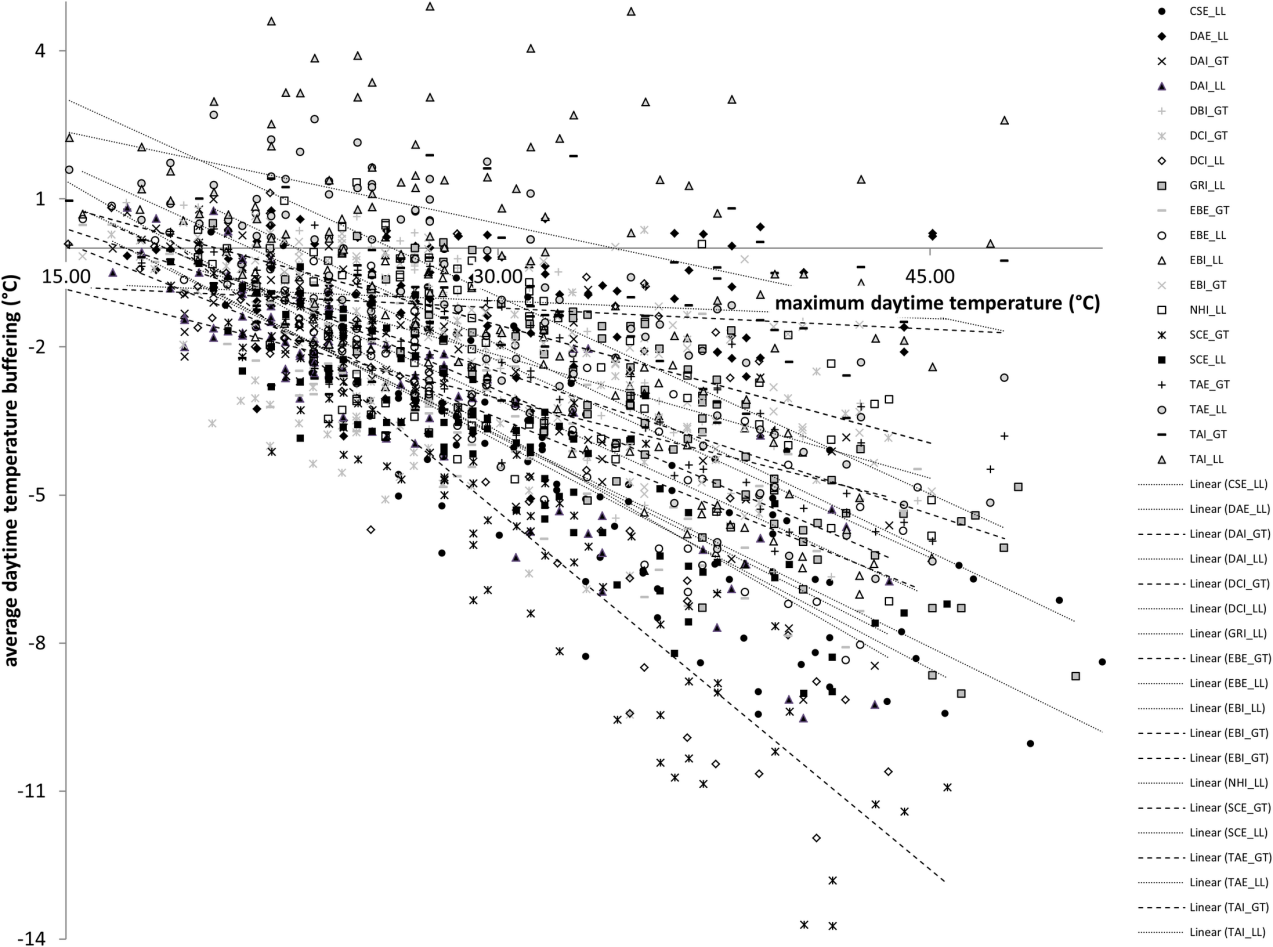
Average daytime temperature buffering (compared to the ground-below-canopy microhabitat in the same location) provided by eight grass-tree (symbols, dashed trendlines) and eleven leaf-litter (shapes, dotted trendlines) microhabitats over 92 days during the 2015/2016 summer in vegetation fragments in the Fleurieu Peninsula. Regression lines were fitted using the method of least squares, assuming a linear relationship. There was no significant difference between the means of the slopes in the two microhabitats (*t* = 0.894; d.f. = 17; *p* = 0.192).

The amount of temperature buffering provided by grass-tree and leaf-litter microhabitats was mostly determined by the maximum temperature of a day, with increasing buffering on hotter days at a rate of about 0.24°C (mean; range = 0.02–0.48°C) of average daily buffering per degree of maximum temperature (Fig 5, Table 5). For the leaf-litter microhabitat, canopy cover was another significant predictor of buffering. These variables explained >63% of the variation in buffering among sites.

**Table 5:** Best linear models explaining average daytime temperature buffering (compared to the ground-below-canopy microhabitat in the same location) for grass-tree and leaf-litter microhabitats, including only significant variables.

	Grass-tree			Leaf-litter		
	*t*-value	*p*-value	Coeff.	*t*-value	*p*-value	Coeff.
Log(Max Temp)	3.87	6.1 × 10^−3^	10.765	4.22	2.0 × 10^−3^	7.19
Log(Canopy)				2.46	0.039	1.14
Intercept	-40.995			-32.51		
Adjusted *r*^2^	0.637			0.735		
*F*-value	15.01 _1,7_			8.16 _2,8_		
*p*-value	6.1 × 10^−3^			2.0 × 10^−3^		

Max Temp = average maximum temperature, Canopy = canopy cover. Significance of variables in linear models was tested at the 5% level using ANOVA of the model including and not including the respective term. Subscripts beside the *F*-value give the degrees of freedom in the numerator and denominator.

## Discussion

Similar to other studies [19,38,39], we found that vegetation cover can reduce surface temperatures. Although canopy cover played a smaller role than the type of microhabitat in moderating temperatures (Table 4), higher canopy cover significantly increased buffering of external temperatures in the leaf-litter (Table 5). Similarly, other studies found that maximum soil temperature can be up to 10°C higher in inter-canopy spaces (compared with under the canopy) in semi-arid woodlands [58]. This further highlights the need to better understand the temperature buffering provided by forests and woodlands [40]. The relationship between canopy cover and temperature buffering should be further investigated to determine its nature, as it is often assumed to be linear [59].

Microhabitats, including leaf litter, cavities formed by senescent grass tree leaves, tree hollows and epiphytes [20,34], have the capacity to considerably buffer hot temperatures. In this study, grass-tree and leaf-litter microhabitats maintained average daytime temperatures up to 5.2°C cooler and average daytime relative humidity up to 28% higher, compared to conditions in the ground-below canopy habitat, during the 2015/16 summer in the Fleurieu Peninsula of South Australia. These habitats are therefore able to maintain environmental conditions closer to the optimum requirements of vertebrates [20,60,61] during extreme heat events and are therefore likely to constitute important refuges for wildlife [26,27,62].

The capacity to buffer hot temperatures increased with increasing external temperatures. A similar trend has been observed under canopy cover [39] and for tree hollows [34]. This trend is linked to greater stability of microclimates in microhabitats (compared to external conditions). Such stability has also been observed in other microhabitats [21,26,63]. The capacity to retain cooler, moister and more stable microclimates is important considering the forecasted increase in temperatures and associated extreme events, particularly in South Australia and Mediterranean ecosystems in general [7,8,41]. While the presence of suitable microhabitats that are ‘heat-resistant’ provides some hope for the survival of wildlife under anthropogenic climate change, we also need to consider the continued persistence of the plant species providing these microhabitats. This is unlikely to be the case for all species, as major vegetation changes are predicted, and indeed already occurring, for Mediterranean regions under anthropogenic climate change [6,7,64].

While fine-scale variation in topography is increasingly considered important for predicting the likely impacts of future climate change [10,11], biotic factors are generally not considered (but see [14,24]). This may be due to the unavailability of fine-scale vegetation data [11], but also due to our limited understanding of the impact that vegetation has in locally moderating the prevalent climate, as most climate stations are set up under standard conditions in cleared areas [40]. However, this study clearly demonstrates the important impact of vegetation on local climates and hence the environmental conditions experienced by wildlife.

A significant impact of fragment area on microclimate has been previously reported [65]. While our finding that larger fragments reduce the amount of daily variation in temperature should be confirmed on a larger scale (with more fragments), it would have important implications for the design of nature reserves (e.g. conservation parks). Several processes could, independently or interactively, explain the observed patterns. Firstly, vegetation acts as a wind break, affecting wind speed and boundary layer mixing, and this could result in a more stable temperature regime. [66]. Furthermore, smaller fragments potentially have different edge structures, which could result in in higher temperature variability [65]. Finally, this phenomenon could be caused by the stabilizing effect vegetation exerts on climates. For example, the clearing of woody vegetation in Western Australia [67] and the Amazon basin [68] is believed to have resulted in local reduction of rainfall and increased temperatures.

Our findings therefore have important implications for landscape and biodiversity management. In order to maintain habitat suitable for wildlife, canopy cover and microhabitat diversity need to be maintained. Fire has the potential to destroy microhabitats [69,70], suggesting the frequency of its use in the management of Mediterranean ecosystems needs to be carefully considered. Furthermore, maximizing canopy cover and the availability of microhabitats at a landscape scale could help to effectively protect wildlife in a warming climate with more extreme temperature events. Finally, evidence is building that bigger vegetation fragments are better at moderating macroclimatic conditions. This may contribute to the SLOSS debate (whether a Single Large Or Several Small reserves are better for protecting biodiversity [71]) in conservation science, as it suggests that larger reserves may be better in retaining more stable microclimates.

## Supporting information

S1 Fig Set up and shielding of microsensors. a) Microsensor as placed in ground-below-canopy microhabitat at about 50 cm height on a bamboo stick in an inverted white plastic cup covered with white duct tape; b) Set up of microsensors placed in leaf-litter and grass-tree microhabitats: placed inside metal tea strainers covered with white duct tape on the upper half; c) and d) Placement of microsensors in leaf-litter and grass-tree microhabitats, respectively–the tea strainer was attached to white thread for easy retrieval and the red arrow indicates entrance point of into microhabitat.
(TIF)

S1 File Plant abundances for each study site. Abundance was determined using a modified Braun-Blanquet scale (5 = >75% cover; 4 = 50–74% cover; 3 = 25–49% cover; 2 = 10–25% cover; 1 = 10% cover) for a single circular plot with a radius of 11.28 m (area = c. 400 m^2^). See text of manuscript for site codes.
(XLSX)

S2 File Temperature raw data for all study sites. See manuscript for site codes. BC = ground-below-canopy microhabitat (0.5 m above the ground), GT = in the cavity formed by the senescent leaves of a grass tree; LL = in the leaf litter.
(XLSX)

S3 File Relative humidity raw data for all study sites. See manuscript for site codes. BC = ground-below-canopy microhabitat (0.5 m above the ground), GT = in the cavity formed by the senescent leaves of a grass tree; LL = in the leaf litter.
(XLSX)
