# Supplementary figures and images for: Microhabitats and canopy cover moderate high summer temperatures in a fragmented Mediterranean landscape
Source: PLoS One. 2017 Aug 14;12(8):e0183106. doi: 10.1371/journal.pone.0183106 (PMC5555690; doi:10.1371/journal.pone.0183106)

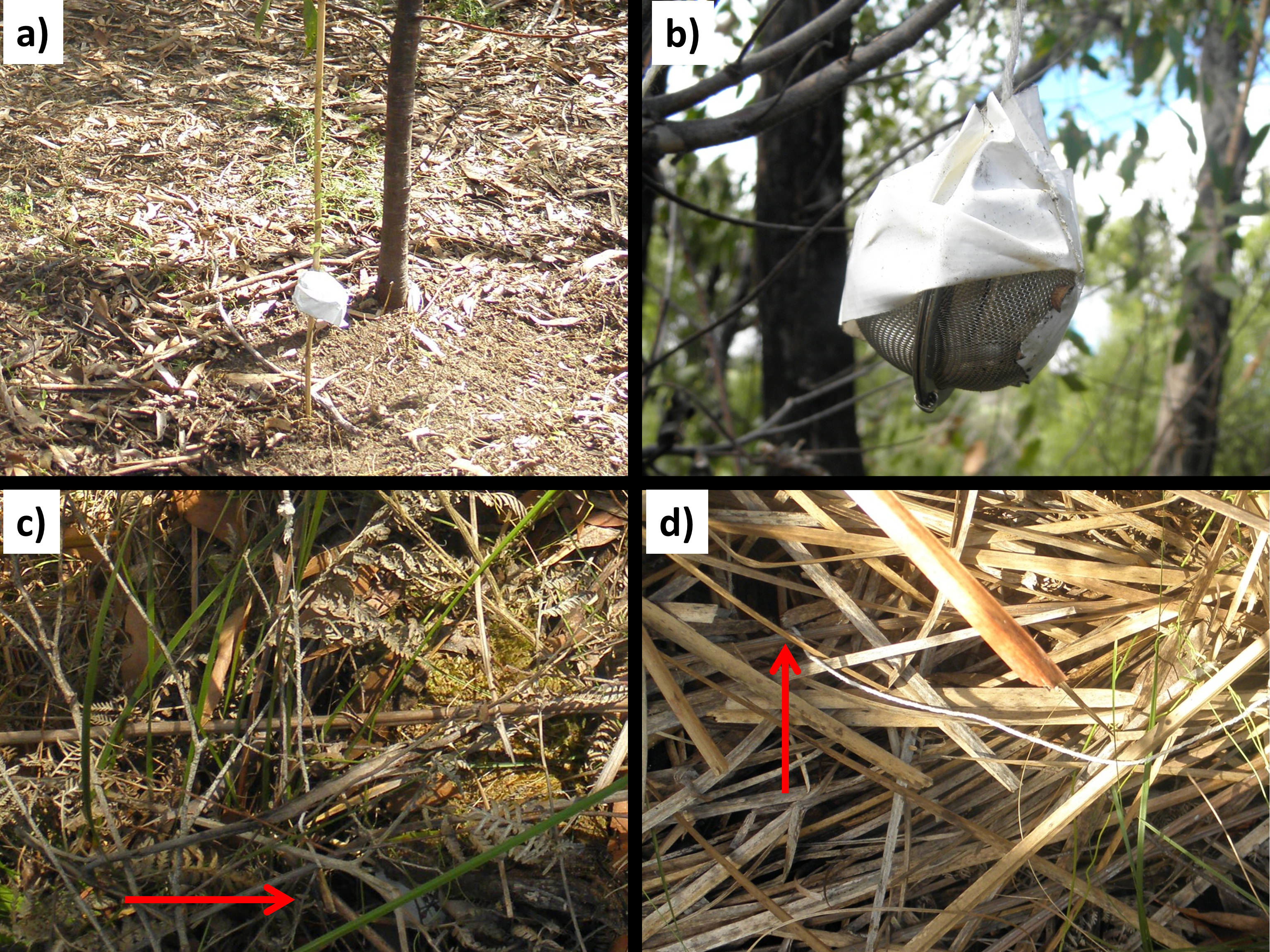

Supplement: S1 Fig — a) Microsensor as placed in ground-below-canopy microhabitat at about 50 cm height on a bamboo stick in an inverted white plastic cup covered with white duct tape; b) Set up of microsensors placed in leaf-litter and grass-tree microhabitats: placed inside metal tea strainers covered with white duct tape on the upper half; c) and d) Placement of microsensors in leaf-litter and grass-tree microhabitats, respectively–the tea strainer was attached to white thread for easy retrieval and the red arrow indicates entrance point of into microhabitat. (TIF) [file pone.0183106.s001.tif]
